# Biocontrol efficacy of cajeput oil against *Anopheles stephensi* L. mosquito and its effect on non-target species

**DOI:** 10.3389/fphys.2024.1357411

**Published:** 2024-03-01

**Authors:** Perumal Vivekanandhan, Tahani Awad Alahmadi, Mohammad Javed Ansari, S. P. Subala

**Affiliations:** ^1^ Department of General Pathology, Saveetha Dental College and Hospitals, Saveetha Institute of Medical and Technical Sciences (SIMATS), Saveetha University, Chennai, India; ^2^ Department of Pediatrics, College of Medicine and King Khalid University Hospital, King Saud University, Riyadh, Saudi Arabia; ^3^ Department of Botany, Hindu College Moradabad (Mahatma Jyotiba Phule Rohilkhand University Bareilly), Moradabad, India; ^4^ Department of Biotechnology, Ayya Nadar Janaki Ammal College (Autonomous), Sivakasi, India

**Keywords:** botanical essential oil, *Anopheles stephensi*, Eudrilus eugeniae, acetylcholinesterase (AchE), α β-carboxylesterase

## Abstract

Chemical insecticides are effective at controlling mosquito populations, but their excessive use can pollute the environment and harm non-target organisms. Mosquitoes can also develop resistance to these chemicals over time, which makes long-term mosquito control efforts challenging. In this study, we assessed the phytochemical, biochemical, and insecticidal properties of the chemical constituents of cajeput oil. Results show that *Melaleuca cajuputi* essential oil may exhibit mosquito larvicidal properties against *Anopheles stephensi* larvae (second-fourth instar) at 24 h post-treatment. At 24 h post-exposure, the essential oil resulted in a significant decrease in detoxifying enzymes. All of these findings indicate that cajeput oil infects *An. stephensi* larvae directly affect the immune system, leading to decreased immune function. Cajeput oil significantly affects the second, third, and fourth instar larvae of *An. stephensi*, according to the bioassay results. Cajeput oil does not induce toxicity in non-target *Eudrilus eugeniae* earthworm species, as indicated by a histological study of earthworms. Phytochemical screening and GC-MS analysis of the essential oil revealed the presence of several major phytochemicals that contribute to mosquito larvicidal activity. The importance of cajeput oil as an effective candidate for biological control of the malarial vector *An. stephensi* is supported by this study.

## Highlights

• M. cajuputi *essential oil* shows *high mosquito larvicidal properties against second to fourth instar* An. stephensi *larvae within 24 h post-treatment.*
• M. cajuputi *essential oil resulted in a decrease in larval detoxifying enzymes. All of these findings indicate that cajeput oil infects* An. stephensi *larvae directly affect the immune system, leading to decreased immune function.*
• M. cajuputi *does not cause toxicity in non-target* E. eugeniae *earthworm species, as indicated by a histological study of earthworms.*

## Introduction

When left uncontrolled, the malarial mosquito, *An. stephensi*, possess a serious threat to human health. This mosquito species is responsible for the spread of parasites that cause malaria in humans (Okoro et al., 2018; Vivekanandhan et al., 2021; Manikandan et al., 2023). Malaria remains a global public health issue, with an estimated 247 million cases and 619,000 deaths worldwide in 2022, primarily affecting children on the African continent. Malaria primarily affects the intertropical region, where climatic and geographical conditions are favorable for the spread of the disease’s vector, the female Anopheles species. A variety of chemical insecticide modes of action have been demonstrated to be effective interventions for mosquito population reduction in the field (Manjarres-Suarez and Olivero-Verbel, 2013). However, repeated application of these chemicals may lead to the development of insecticide-resistant mosquito populations, causing product failures and reducing effectiveness (Vivekanandhan et al., 2021). It is critical to develop new alternative methods for reducing mosquito populations, which helps protect human health.

Chemical constituents derived from microbes, plant crude extract and essential oils (EOs) are promising for reducing mosquito populations (Vivekanandhan et al., 2018a; Vivekanandhan et al., 2018b; Vivekanandhan et al., 2018c; Balumahendhiran et al., 2019; Logeswaran et al., 2019; Vivekanandhan et al., 2020a; Vivekanandhan et al., 2020b; Perumal et al., 2023a; Perumal et al., 2023b; Perumal et al., 2023c). In the case of insecticide-resistant mosquitoes, EOs can act as biorational alternatives or synergist additives to existing insecticides. Recent studies have already reported on the insecticidal activities of essential oils (EOs) and their ability to enhance the effectiveness of insecticides (Amer and Mehlhorn, 2006a; Norris et al., 2018; O'Neal et al., 2019). Many species in the Myrtaceae family plants are being studied as potential bioinsecticides (Pratiwi and Nurlaeni, 2021). Melaleuca leucadendron, Melaleuca quinquenervia, and M. cajuputi have been reported as adulticides, repellents, and growth regulators for mosquitoes (Amer and Mehlhorn, 2006b; Noosidum et al., 2008; Bakar et al., 2012; Brophy et al., 2014; Leyva et al., 2016). M. cajuputi is a native of Southeast Asia and Australia. In Southeast Asia, it is considered a common household medicine with a few medicinal applications. Now, US Food and Drug Administration (FDA) approved cajeput oil has an edible use because it is non-toxic and non-sensitizing.

Insect acetylcholinesterase (AChE) is an important enzyme in the nervous system that facilitates nerve signal transmission by degrading acetylcholine. Pesticides frequently inhibit AChE activity and cause neurotoxic effects in insects (Vivekanandhan et al., 2022; Perumal et al., 2023a; Perumal et al., 2023b; Perumal et al., 2023c). Understanding AChE offers insights into insecticide mechanisms and aids in the development of pest control strategies. Insect α and β carboxylesterase enzymes play critical roles in the detoxification and metabolism of xenobiotics, especially insecticides. These enzymes are found in the digestive system and other tissues of insects and are used to catalyse the hydrolysis of ester-containing compounds. Insects can break down and eliminate a wide range of exogenous chemicals, including synthetic pesticides. The importance of these enzymes in insecticide resistance mechanisms, where increased expression or mutations in their genes can confer resistance to specific chemical agents. Understanding the biochemical pathways involving α and β carboxylesterases is critical for developing long-term pest control strategies. Nowadays, Researchers perform AchE enzyme levels in order to decipher the mechanisms used by insects to counteract the effects of insecticides, which will ultimately aid in the design of more effective and environmentally friendly pest control approaches that reduce the risk of resistance development in target insect populations.

E. eugeniae, also known as the African nightcrawler, is a species of earthworm that is widely used in vermicomposting due to its efficient organic matter decomposition. Because of its sensitivity to environmental changes, E. eugeniae serves as an ecological indicator (Perumal et al., 2023a; Perumal et al., 2023b). Monitoring E. eugeniae populations helps assess soil quality because earthworms play an important role in soil health. Changes in behaviour can indicate ecological shifts, assisting in the assessment of environmental conditions (Vivekanandhan et al., 2023). The aim of this study was to extract essential oil and test the bio-insecticidal activity of cajeput oil against malarial mosquito larvae, An. stephensi, using toxicological, biochemical, and non-target species toxicity approaches. These findings shed light on cajeput oil’s bio-insecticidal activity against malarial mosquito larvae, *An. stephensi*, and suggest that it could be used as a bioinsecticide to reduce mosquito populations and ultimately, prevent community transmission of mosquito-borne pathogens that cause human disease.

## Materials and methods

### Plant materials

**FIGURE 1:**
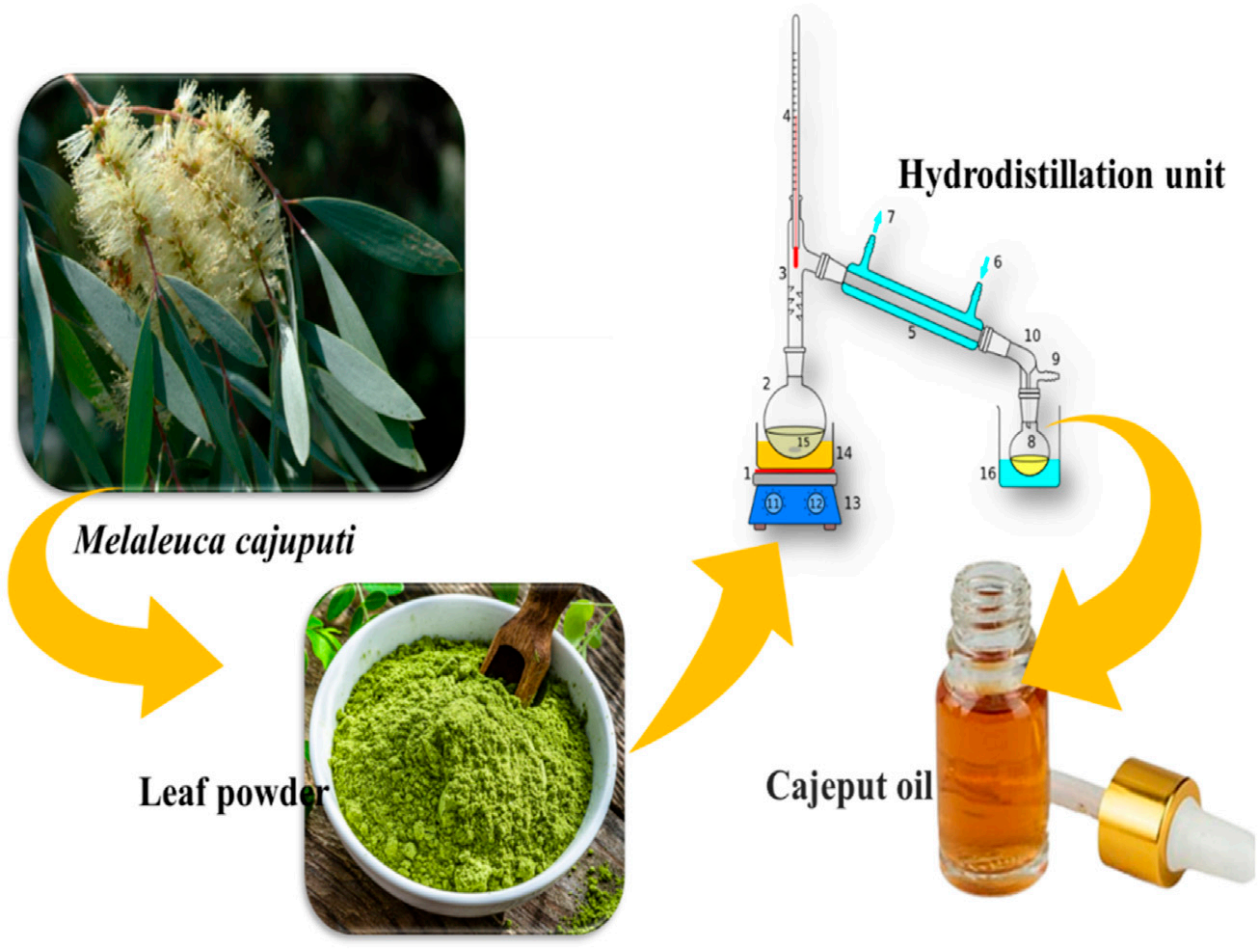
The schematic diagram illustrates the process of extracting essential oil from *Melaleuca cajuputi*.

*M. cajuputi* leaves (Figure 1) were collected in Tamil Nadu (11° 7′ 37.6428″ N and 78° 39′ 24.8076″ E). Plant taxonomists identified the botanical nomenclature of the plant, prepared the herbarium specimen (VK_325), and stored it in the entomological laboratory.

### Plant materials preparation and essential oils extraction

Collected leaves of the *M. cajuputi* plant were washed with tap water to remove surface particles and debris before being ground into powder. The essential oil was then extracted using hydrodistillation methods with a modified Clevenger apparatus, as described by Vivekanandhan et al. (2018c). In each instance, 100 g of leaf powder was distilled for 3 h in a 500 mL flask containing 300 mL of distilled water. The essential oil was extracted from the leaves and collected in sterilized glass vials. To remove water traces, anhydrous sodium sulfate was used, and the *M. cajuputi* essential oil samples were stored at 4°C for future experiments.

### Mosquito culture

The larvae of *An. stephensi* mosquitoes were obtained from the National Centre for Disease Control (NCDC) in Mettupalayam, Tamil Nadu, India. Mosquito larvae were reared and maintained in a laboratory at a temperature of 27°C ± 2°C and a humidity of 75%. The larvae were fed a mixture of dog biscuits, millet powder, and yeast powder in a 3:3:1 ratio, as previously described (Vivekanandhan et al., 2018a).

### Larvicidal bioassay

The larvicidal bioassay of *M. cajuputi* essential oils was conducted following the World Health Organization method (WHO, 2005; Pratheeba et al., 2019). About 30 s to fourth instar larvae were individually transferred into bioassay trays containing 249 mL of tap water and 1.0 mL of the specified plant essential oil at various concentrations (30, 50, 100, 150, and 200 ppm/mL) dissolved in 1 mL of 0.5% dimethyl sulfoxide (DMSO). Control groups were treated with DMSO alone. Larval mortality was calculated 24 h after treatment. Each concentration has five replicates, with 30 larvae in each replicate.

### Larval enzyme preparation

*An. stephensi* larvae from both the control group and the group treated with essential oil were selected for studying detoxification enzyme activity 24 h after treatment, specifically in the fourth instar larvae. The larval midgut was dissected, homogenized using 2 mL of phosphate-buffered saline, and then centrifuged at 10,000 rpm for 15 min at 4 °C. The supernatant was then decanted into a clean Eppendorf tube, placed on ice, and used immediately for subsequent enzyme assays.

### Acetylcholinesterase assay

Ellman et al. (1961) utilized acetylcholine iodide as a substrate to test the acetylcholinesterase activity of larval homogenate. 50 mL of larval tissues (pH 7.5) were mixed with 850 mL of 100 mM sodium phosphate buffer. Each reaction mixture consisted of 50 mL of 10 mM 5,5′-dithiobis-2-nitrobenzoic acid (DTNB) and 50 mL of 12.5 mM cholinergic iodide, both of which were kept at room temperature for 5 min. The sample’s optical density was measured at 405 nm using an appropriate blank and a Multiskan EX (200–240 V) spectrophotometer.

### Carboxylesterase assays

Alpha and beta-carboxylesterase enzyme activity was assessed using larval midgut homogenate as described by Van Asperen (1962). 30 mL of homogenate was mixed with 1 mL of sodium phosphate buffer (pH 7.0, 100 mM) containing 250 mM of α- and β-naphthyl acetate and incubated at room temperature for 30 min. To inhibit the enzymatic process, each reaction mixture was treated with 40 mL of 0.3% Fast Blue B in 3.3% sodium dodecyl sulfate (SDS). The mixture was left at room temperature for 15 min to develop its distinct color. The optical density was measured using a Multiskan EX-200–240 V spectrophotometer (Thermo Scientific) and the corresponding reagent or blank. α- and β-carboxylesterase enzyme activities were determined using a standard curve with naphthol as a control.

### Earthworm non-target toxicity

*E. eugeniae*, also known as the “African nightcrawler,” was sustained in the laboratory by using cow dung. Vivekanandhan et al. (2023) cultured earthworms at a constant temperature of 26°C ± 2°C. The contact filter paper test involved placing a section of filter paper on a 5-cm petri plate and evaluating different concentrations of essential oils (30, 50, 100, 150, and 200 ppm), as well as monocrotophos (200 ppm; a commercial pesticide) using Sigma-Aldrich Pestanal^®^ analytical standard. Earthworms were transferred individually onto the treated paper tissue, which was moistened with 2 mL of sterile distilled water (ddH2O). The treatments were kept in the dark at 25°C ± 1 °C for 24 h, and the mortality rate was assessed. Similarly, artificial soil composed of 16% kaolinite clay, 72% fine sand, and 12% ground sphagnum peat was used in artificial soil tests (ASTs). The tests were then analyzed to determine the mortality rate at different time points, specifically 24 h. The control group of earthworms was administered with PBS saline alone, and the entire treatment was carried out with five replicates.

### Gut histochemistry

The study investigated the impact of essential oils on the midgut tissues of earthworms and fourth-instar larvae at sub-lethal doses. Mosquito larvae and earthworm samples from the mid-gut control group and those treated with a sub-lethal dosage (200 ppm) were immobilized overnight in Bouin’s chemical reagent. The sectioned blocks were then cooled to approximately 26°C ± 2°C for 2 h before being cut into 1.5 mm portions using a cryo-microtome (Cryocut 1800; Leica, Germany). The entire mid-gut was sectioned for microscopic slides and observed under an Optika Fluo Series HBO (mercury short-arc lamp) light microscope (Murfadunnisa et al., 2019).

### Phytochemical screening

Preliminary phytochemical analysis was conducted on essential oils extracted from *M. cajuputi* using methods slightly modified from Satheesh and Wesley, (2012).

### Alkaloids

**Mayer’s/Bertrand’s/Valser’s test:** One mL of essential oil was mixed with 1 mL of con. Add HCL, followed by two to three drops of Mayer’s reagent. The presence of alkaloids is indicated by the formation of a creamy white or yellow precipitate (Auwal et al., 2014).

### Flavonoid

**Lead acetate test:** The researchers utilized a colorimetric assay to quantify the overall flavonoid content (Auwal et al., 2014). They added 100 μL of extract to 4 mL of distilled water, followed by the addition of 0.3 mL of 5% sodium nitrite and 0.3 mL of 10% aluminum chloride after 5 min. Then, 2 mL of 1 M sodium hydroxide was added to the mixture after 6 min. The mixture was promptly diluted with 3.3 mL of distilled water and thoroughly mixed. The absorbance was then measured at 510 nm against a blank using catechin as the standard for the calibration curve.

### Phenol

**Ferric chloride test:** One mL of essential oil was combined with a 5% ferric chloride solution. The presence of phenol is confirmed by the formation of a reddish-brown precipitate (Pandey et al., 2014).

### Saponin

**Foam test:** One mL of essential oils was mixed with 1 mL of distilled water. The solution was then vigorously shaken, and the formation of foam indicated the presence of saponin (Dhawan and Gupta, 2017).

### Quinones

**Conc. HCl test:** One milliliter of essential oils was mixed with 0.5 mL of concentrated hydrochloric acid (HCl). The formation of a yellow precipitate indicates the existence of quinones (Maria et al., 2018).

### Tannin

**Lead sub acetate test:** 3 drops of lead subacetate solution were mixed into 5 mL of plant essential oils dissolved in 45% ethanol. The presence of tannins is demonstrated by the appearance of a creamy, viscous precipitate (Sheel et al., 2014).

### GC–MS analysis

GC-MS analysis was conducted to identify anti-ulcer compounds in the essential oil. For the sample preparation for GC-MS analysis, a 5 MSTG column (30 m × 0.25 mm, 0.25 µm) and a THERMO TRACE 1300 gas chromatograph-mass spectrometer coupled with the TSQ-8000 mass spectrometer were utilized. As a carrier gas, helium was used at a flow rate of approximately 1 mL/min. The oven’s temperature ranged from 60°C to 280°C, increasing at a rate of 10°C per minute. With a splitting ratio of 1:10 and a temperature of 250 °C, the injection volume was set to 1.0 L. The mass spectrum was captured for 31 min at an ionizing energy of 70 eV, with the mass spectrometry transfer line temperature maintained at 280 C and the ion source temperature maintained at 230 C, respectively (Geetha et al., 2013).

## Results

### Larvicidal bioassay

**FIGURE 2:**
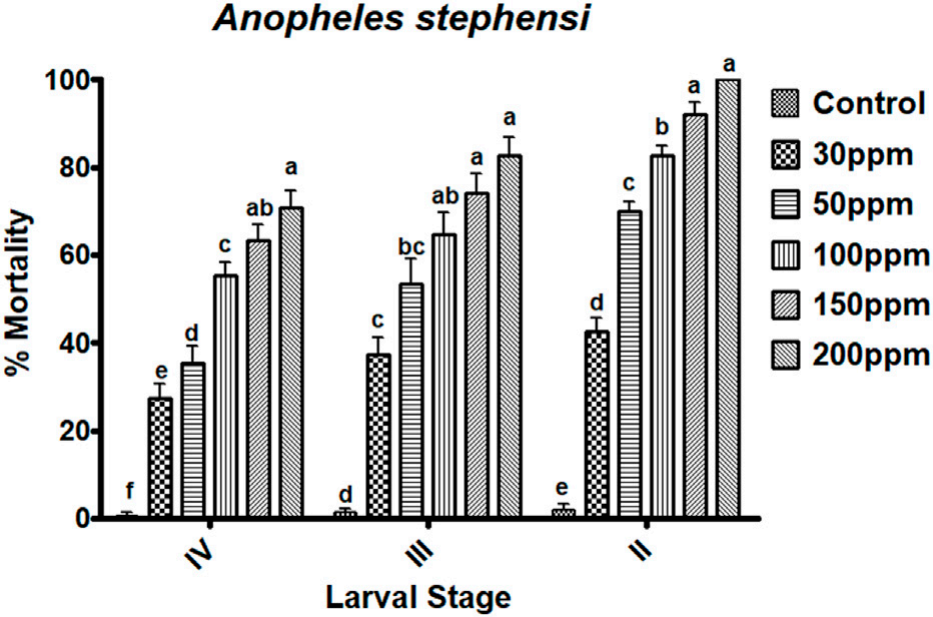
*Melaleuca cajuputi* leaf essential oils have larvicidal activity against malarial vector *An. stephensi* larvae in the II, III, and IV instars. In a Tukey test, means ((SE) standard error) followed by the same letters above bars indicate no significant difference (*p* ≤ 0.05).

**FIGURE 3:**
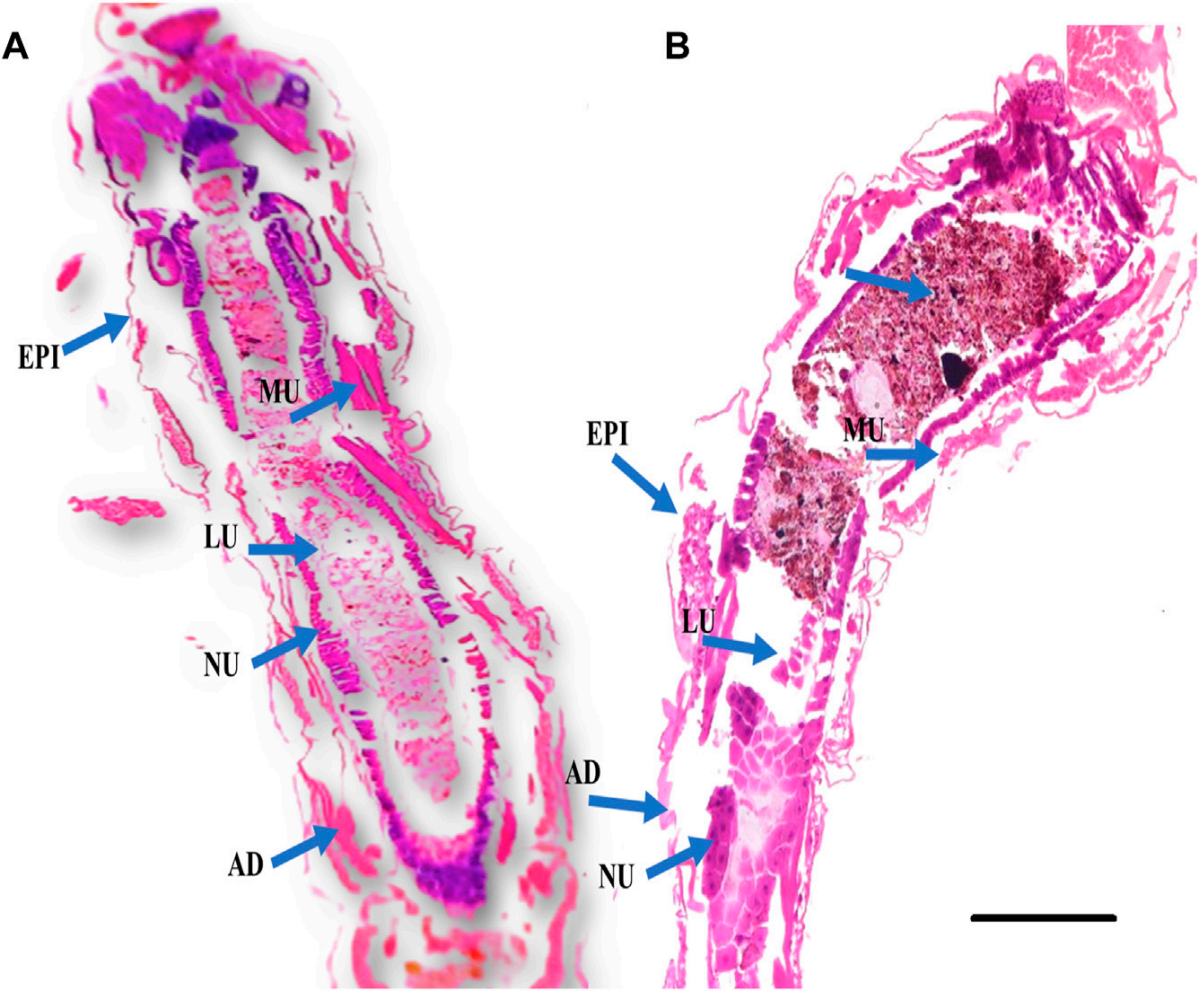
Histological cross sections of fourth instar larvae of *An. stephensi* at 24 h post treatment with plant essential oil showed significant damage to the larval gut tissues. **(A)** Control larvae (without essential oil treatment); **(B)** Larval tissues of essential oil-treated larvae are abnormal compared to the control group, exhibiting vacuolated gut epithelium (epi), muscles (mu), nucleus (nu), and fat body (fb). The gut lumen (lu) and adipose tissue (ad) show brown granules, indicating fragmentation or damage caused by the essential oil. The scale bar size is 300 µm.

The essential oils of *M. cajuputi* had a dose-dependent mortality rate against all instar stages of *An. stephensi* mosquito larvae (second-fourth). The mortality percentage of *An. stephensi* was no significant across treatment and control, with the rate of mortality being prominent at the maximum dosage (200 ppm) in the second 100% (F _*(5,12)*_ = 304.147, *p* ≤ 0.01), the third 82.66% (F _*(5,24)*_ = 45.192, *p* ≤ 0.01), and the fourth instar 70.66% (F_*(5,24)*_ = 62.229, *p* ≤ 0.01) (Figure 2; Figure 3). Furthermore, the lethal concentration (LC_50_ and LC_90_) of essential oils on *An. stephensi* were determined at the second instar (34.663 and 114.863 ppm/mL), third instar (48.848 and 392.088 ppm/mL), and fourth instar (84.226 and 671.625 ppm/mL) respectively (Figure 2; Figure 3).

### Larval biochemical assay

#### Acetylcholinesterase assay

**FIGURE 4:**
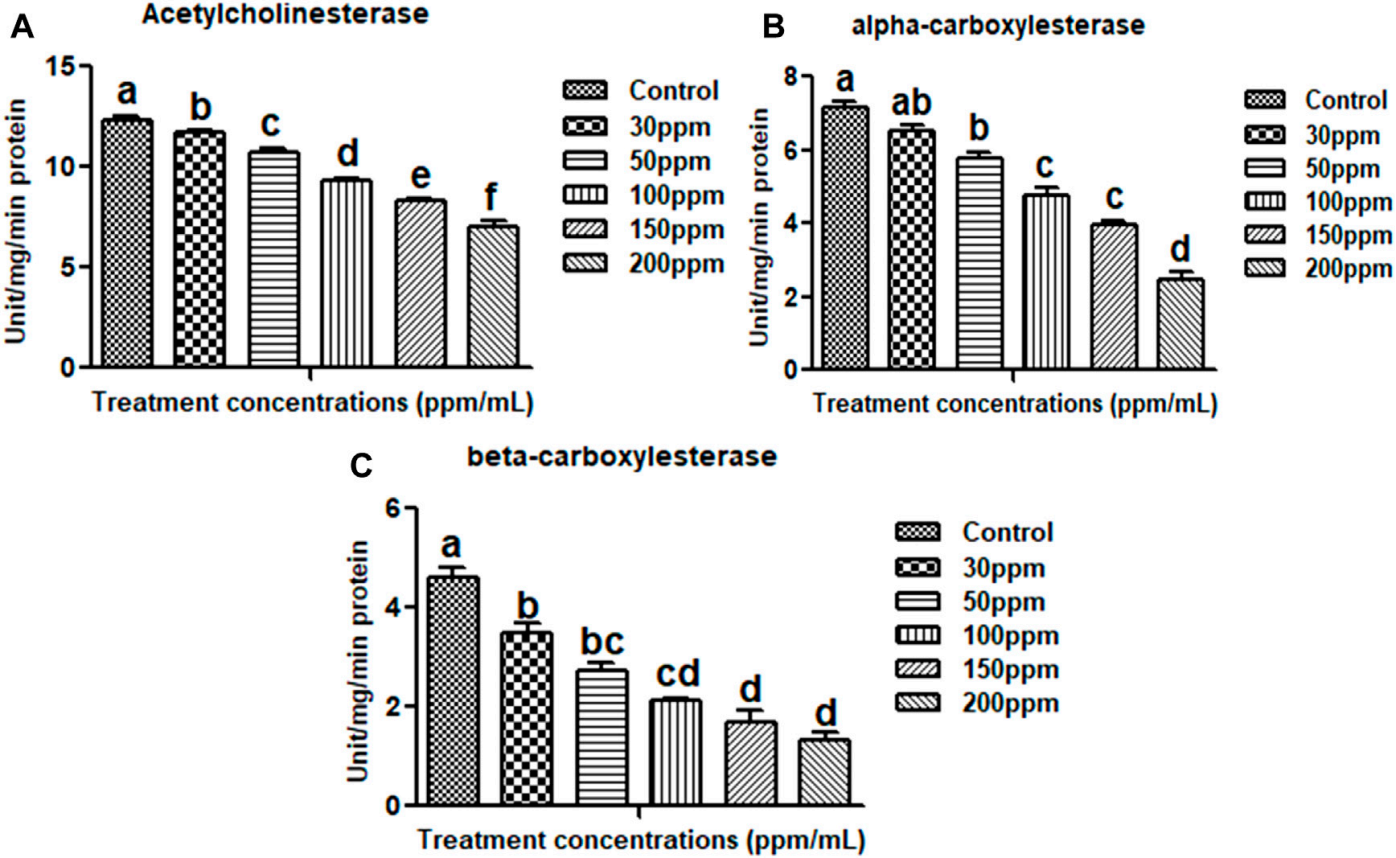
Acetylcholinesterase **(A)**, alpha carboxylesterase **(B)**, beta carboxylesterase **(C)**. There is no statistically significant difference between values that follow the same letter (one-way ANOVA), as shown by the Tukey test at *p* = 0.05. Notes: the mid-gut tissues of larvae were stained with ehrlich’s haematoxylin and viewed under a light microscope at ×100 magnification.

The biochemical effects of essential oils from *M. cajuputi* on *An. stephensi* larvae were investigated. The lowest AchE enzyme activity was found to be in higher test concentration (200 ppm/mL). When larvae were exposed to essential oils from *M. cajuputi*, their acetylcholinesterase enzyme activity was significantly (*p* < 0.05) reduced compared to controls (12.33–7.0 mg protein/mL of homogenate) (df 5; F (_*5,12)*_ = 0.728; *p* ≤ 0.01) (Figure 4A). The enzyme level was found to be normal in the control larvae. In the treatment the expression of AchE enzymes was primarily dose-dependent, with larvae exposed to essential oils from *M. cajuputi* exhibiting lower levels. The amount of AchE enzyme activity was decreased when essential oil concentrations are increased (Figure 4A).

#### α -carboxylesterase and β-carboxylesterase assay

Results showed that *M. cajuputi* essential oils on *An. stephensi* larvae resulted in lower levels of α and β-carboxylesterase enzyme activity (Figures 4B,C), after 24 h of treatment. The essential oils of *M. cajuputi* had a dose-dependent effect on the enzyme activity of α-carboxylesterase in larvae. When larvae were exposed to essential oils from *M. cajuputi*, their α-carboxylesterase activity was not significant compared to controls (7.16–2.45 mg protein/mL of homogenate) (df 5; F_*(5,12)*_ = 99.947; *p* ≤ 0.01) (See Figure 4B). A similar finding was made with β-carboxylesterase, which showed a significant decrease in activity (from 5.0 to 1.1 mM/protein/mg/min) in *An. stephensi* larvae (df = 5; F_*(5, 12)*_ = 56.278; *p* ≤ 0.01) (See Figure 4C).

#### Non-target toxicity assay

**FIGURE 5:**
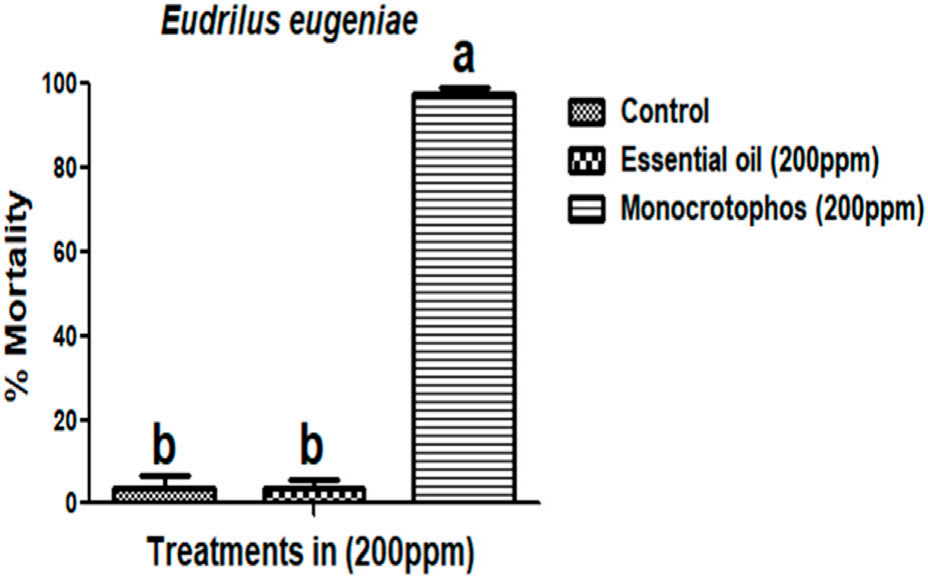
*Melaleuca cajuputi* essential oils minimal toxicity effects on *Eudrilus eugeniae* compared to chemical pesticide monocrotophos. In a Tukey test, means ((SE) standard error) followed by the same letters above bars indicate no significant difference (*p* ≤ 0.05).

**FIGURE 6:**
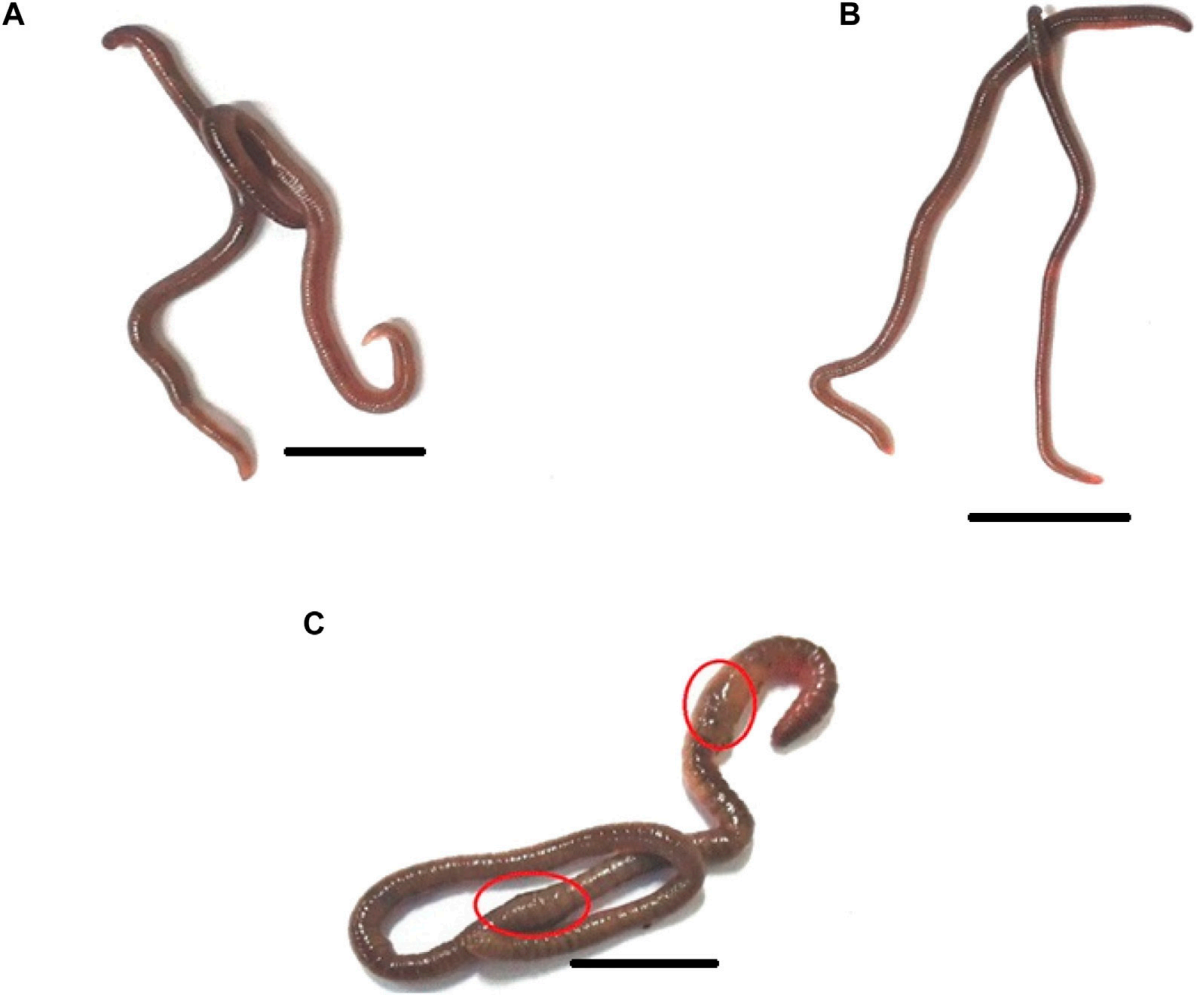
The morphology of the earthworm *Eudrilus eugeniae* in response to distilled water (negative control), essential oil, and Monocrotophos (positive control). Both the control **(A)** and essential oil treatments had normal external morphology **(B)**. Monocrotophos-treated treatments have abnormal morphology **(C)**. Chemical pesticide exposure altered the morphology of earthworms, affecting their body structure and size. The red circle indicates that chemical pesticides damaged the earthworm’*s epidermis*. The scale bar size is 10 mm.

M. cajuputi essential oils had a minimal mortality rate were observed on E. eugeniae earthworm species. The toxicity of M. cajuputi-derived essential oils was less or minimal at the maximum dosage (200 ppm), which showed 4% mortality. This was similar to the control treatment as compared to the market-available chemical pesticide monocrotophos (200 ppm) showed 97.33% mortality (F (1,8) = 890, *p* ≤ 0.01) (Figure 5; Figure 6). All treatment dosages are significant (*p* < 0.05) in comparison to the control, but there is no significant difference between the control and chemical pesticide treatments (Figure 5; Figure 6).

#### Gut histochemistry

**FIGURE 7:**
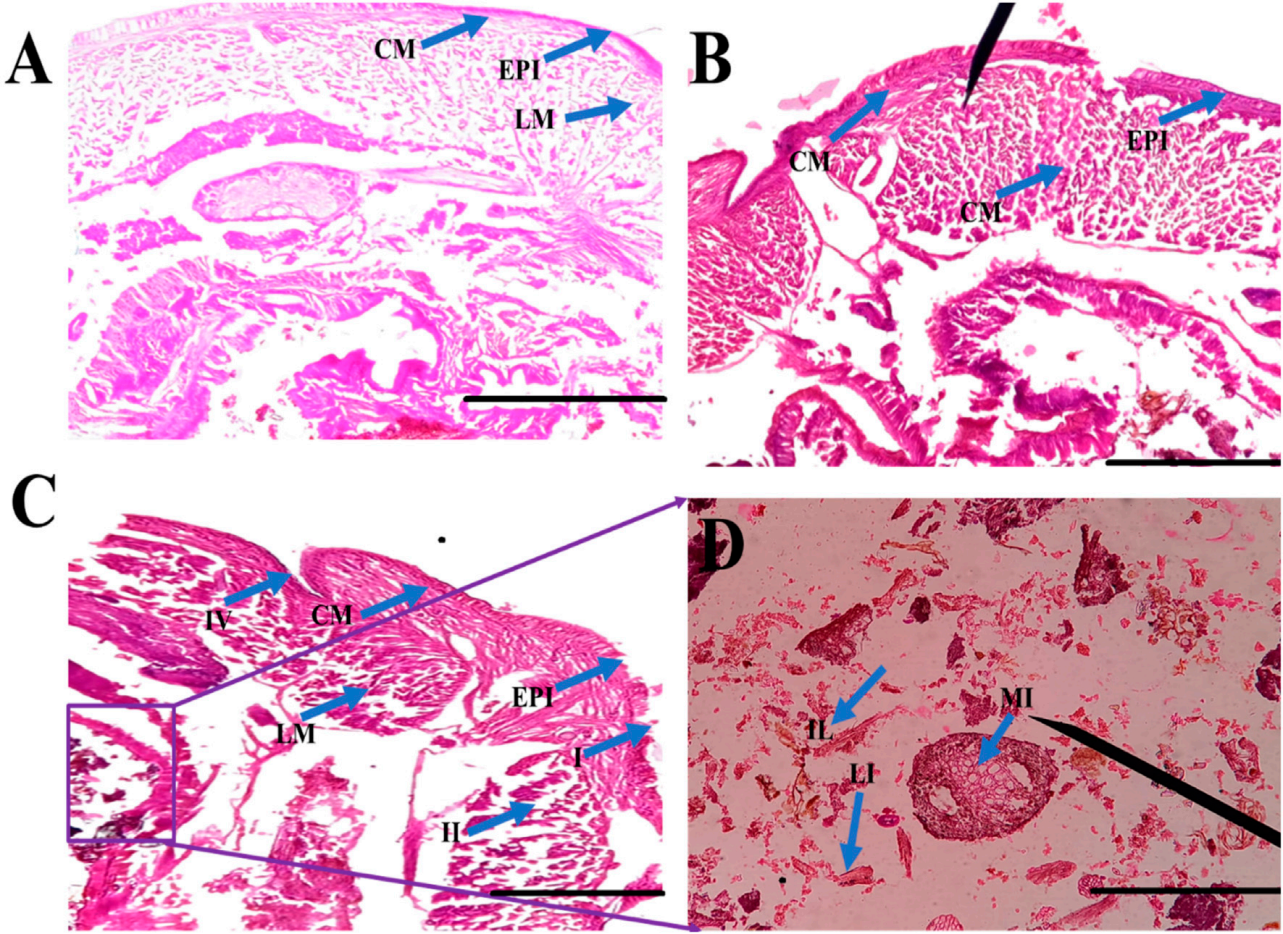
Histological studies of *Eudrilus eugeniae* after 24 h of essential oil treatment revealed that minimal toxicity effects was observed in the essential oil treatment. In gut tissues, no sublethal effects of oils were observed. The histological studies revealed essential oil-treated earthworm gut cells similar to the negative control. In the control and oil treated gut tissues there is no cellular debris in the earthworm gut tissues, and the nuclei are round in shape. Similar outcomes were seen in the control treatment as well. The chemical insecticide monocrotophos (positive control) caused significant damage to gut tissues. (EPI = epidermis, SE = setae, IL = intestinal lumen, LM = longitudinal muscle, CO = coelom, CM = circular muscle, MI = mitochondrion). The scale bar size is 0.1 mm.

*E. eugeniae* is a suitable candidate as a bioindicator in soil toxicology. No deaths occurred within 24 h after essential oil treatment. There were no sublethal effects of essential oils on gut tissues, but the gut tissues of mosquito larvae were highly damaged by the botanical-derived essential oils (see Figure 7). Monocrotophos, a chemical pesticide used as a positive control, induced changes in earthworm gut cells and an irregular epithelial surface. Cellular debris was present, and the nuclei were not spherical (see Figure 7). Figure 4 and Figure 5 show that the essential oil treatment produced the same results as the control.

#### Essential oil phytochemicals

Phytochemical analysis of essential oils extracted from *M. cajuputi* indicates the presence of phenols, flavonoids, alkaloids, quinones, saponins, and tannins (Table 1). The phenols, flavonoids, alkaloids, quinones, saponins, and tannins extracted from *M. cajuputi* essential oils may contribute to the mosquito larvicidal activity (see Table 1).

**TABLE 1:** Qualitative Analysis of the phytoconstituents present in *Melaleuca cajuputi* derived essential oils.

S.No	Phytoconstituents	Test method	Observation	Result
1	Alkaloids	Mayer’s/Bertrand’s/Valser’s test	creamy white or yellow precipitate	Positive
2	Flavonoid	Lead acetate test	creamy white or yellow precipitate	Positive
3	Phenol	Ferric chloride test	formation of a reddish-brown precipitate	Positive
4	Saponin	Foam test	formation of foam	Positive
5	Quinones	Conc. HCl test	formation yellow precipitate	Positive
6	Tannin	Lead sub acetate test	appearance of a creamy, viscous precipitate	Positive

#### GC-MS chemical constituents’ analysis

GC-MS analysis of M. cajuputi essential oils shows the presence of thirty-seven phytochemical constituents. Among the chemical constituents, the nine major are: 1,8-cineole (52.83%); limonene (13.50%); beta-pinene (5.43%); alpha-terpineol (4.65%); alpha-pinene (4.61%); gamma-terpinene (3.48); and beta-caryophyllene (3.11%). These major chemicals may have larvicidal activity (Table 2).

**TABLE 2:** GC-MS analysis of *Melaleuca cajuputi* leaves derived essential oils.

S.No	RT mins	Area %	Compound Name	Structure	Biological activity
1	5.25	4.61	alpha-Pinene	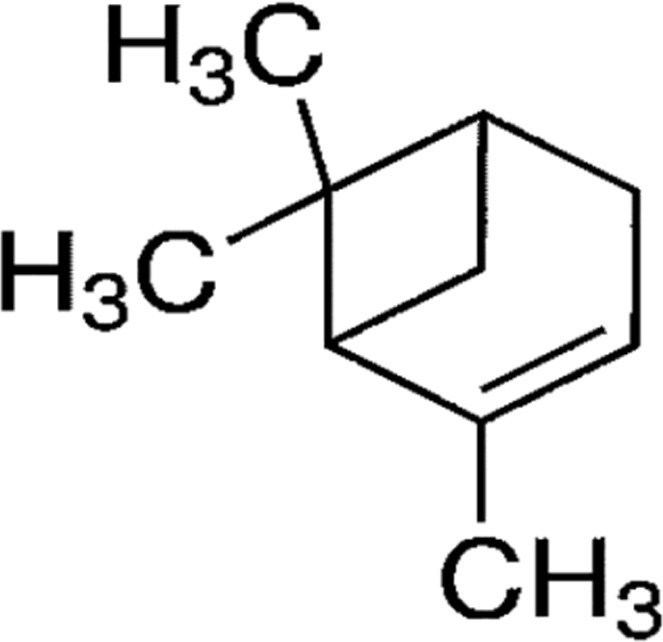	Treatment of bladder, kidney stones, antimicrobial, anti-inflammatory
2	8.06	2.34	alpha-Thujene	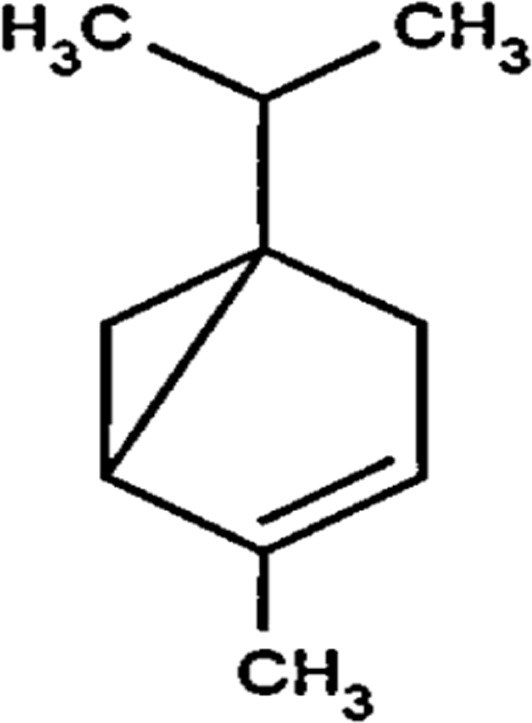	Cosmetic and fragrance industries, inflammatory disorders such as osteoarthritis, bronchial asthma, chronic colitis
3	9.36	5.43	beta-Pinene	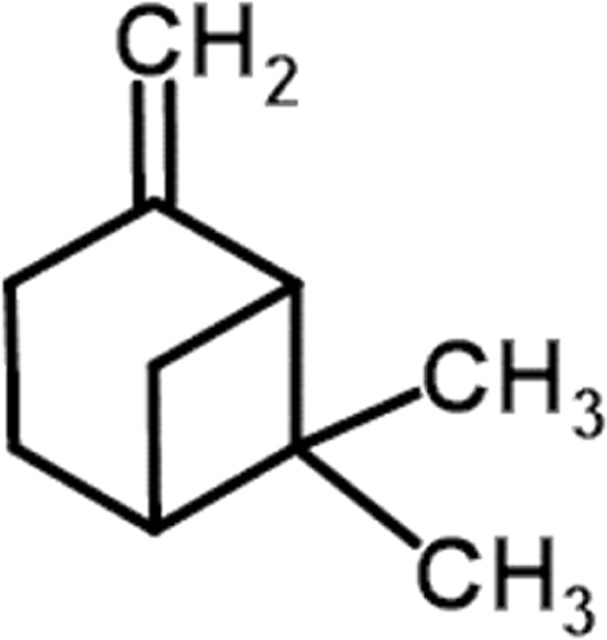	Antibacterial, antidepressant, cytotoxic, and antimicrobial
4	10.62	13.50	Limonene	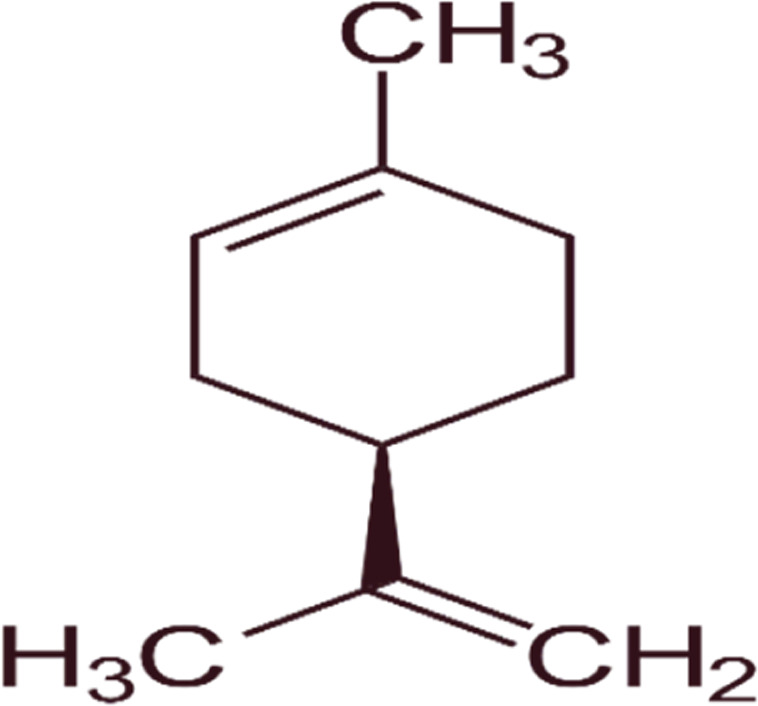	Used in perfumes, household cleaners, foods, and medicines
5	21.49	2.86	delta-3-Carene	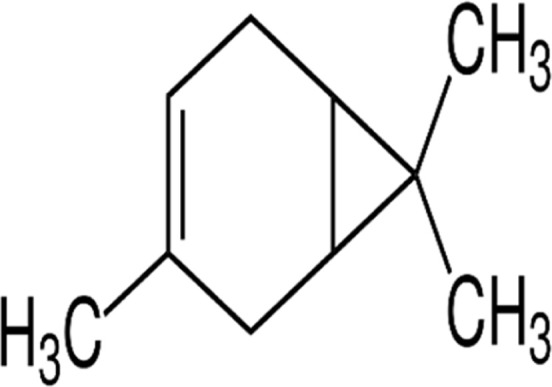	Relieve inflammation related to arthritis or fibromyalgia
6	24.50	52.51	1,8-Cineole	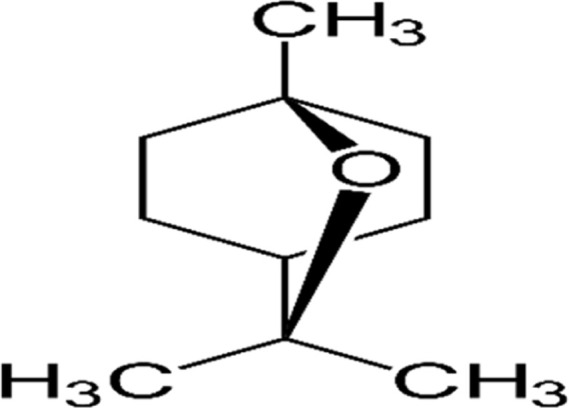	Acute bronchitis, asthma, COPD and sinusitis
7	25.42	3.85	gamma-Terpinene	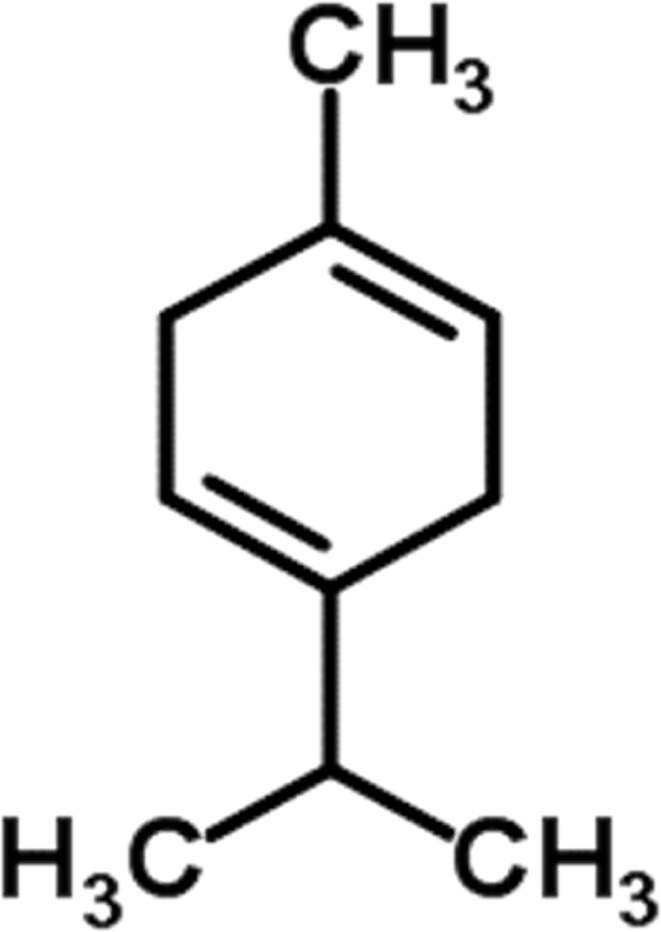	Used in food, flavours, soaps, cosmetics, pharmaceuticals, tobacco, confectionery, and perfume industries
8	32.13	2.52	Terpinolene	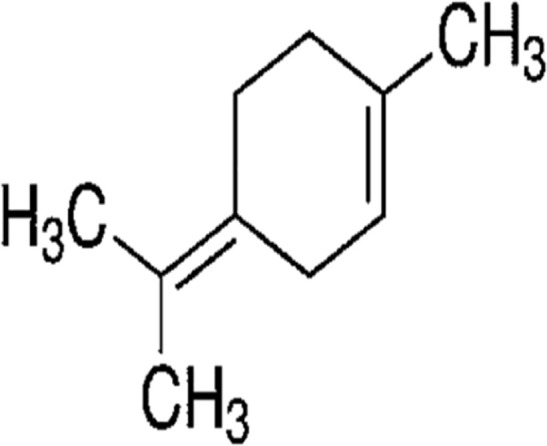	Used for these aromatic qualities in soaps, perfumes, and some insect repellents
9	36.25	4.23	alpha-Terpineol	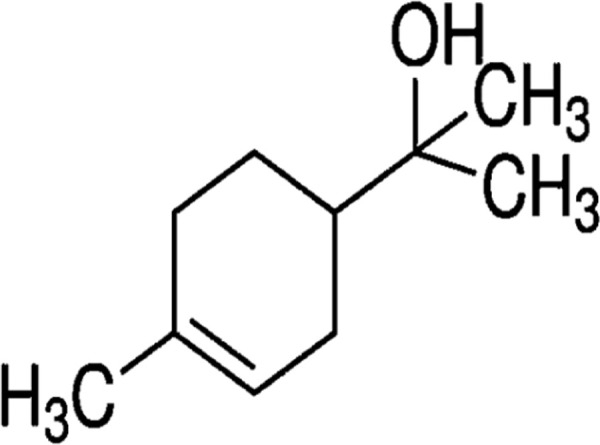	Medicine to strengthen memory and treat dementia

## Discussion

In this study, we focused on the larvicidal properties of *M. cajuputi* plant essential oils against the malarial vector *An. stephensi*. Many researchers have conducted studies on the biological activities of plant essential oils. *M. cajuputi* is a native plant of Australia and belongs to the Myrtaceae family (Southwell and Lowe, 1999). It is commonly found in marshy areas near the coast of Malaysia. For centuries, the Aboriginal people of Australia have used the leaves for a variety of medicinal purposes. Locals in Asia have traditionally used its oil to treat joint pain, stiffness, and rheumatism.

The essential oils of *M. cajuputi* had a dose-dependent mortality rate against all instar stages of *An. stephensi* mosquito larvae (second-fourth). The mortality percentage of *An. stephensi* was significant across treatment and control, with the rate of mortality being prominent at the maximum dosage (200 ppm) in the second 100%, the third 82.66%, and the fourth instar 70.66% (Figure 2; Figure 3). Researchers tested cajeput oil (*M. cajuputi*) on *Aedes* mosquito species, which showed high mosquitocidal activity against larvae and adult mosquitoes (Bakar et al., 2012; Bakar et al., 2019). A similar study by Jayakumar et al. (2016) found that eucalyptus essential oil was the most effective against Cx. quinquefasciatus larvae and pupae, with LC50 values of 186.77 mg/L and 206.08 mg/L, respectively. Similarly, M. cajuputi essential oil has a lower LC50 of 120.99 mg/L against Ae. aegypti larvae (Bakar et al., 2019). The present study clearly shows that *M. cajuputi*-derived essential oil had lower LC_50_ and LC_90_ values against larvae of *An. stephensi* were, 34.663 ppm/mL and 114.863 ppm/mL, respectively (Figure 2; Figure 3). Previous research on the effectiveness of *M. cajuputi* (Family: Myrtaceae) essential oil against dengue vectors revealed remarkable mosquito larvicidal and adulticidal activity (Bakar et al., 2012). In general, essential oil act as contact insecticides, with a neurotoxic mode of action that targets GABA and octopamine synapses, as well as acetylcholinesterase (Regnault-Roger et al., 2012). Although the mode of action of essential oils in mosquito larvae is unknown, previous research has shown that plant chemical constituents initially affect the midgut epithelium, gastric caeca, and Malpighian tubules in mosquito larvae (Rey et al., 1999). Similarly, Myrtaceae plant essential oils have larvicidal properties against the dengue vector *Ae. aegypti* larvae (Dias et al., 2015). Sosan et al. (2001) found that essential oils of *Ocimum gratissium*, *Cymbopogon citrus*, and *A. conyzoides* had 100% mortality against *Ae. aegypti* at 120, 200, and 300 ppm concentrations, respectively. Many recent studies have identified plant extracts and essential oils as important sources of botanical insecticides. Plant compounds, rather than synthetic insecticides, may lead to the development of effective natural mosquitocidal products. Because of the volatile nature of plant essential oils, their insecticidal products degrade quickly, posing a lower environmental risk than synthetic insecticides.

The results showed that the biochemical effects of essential oils derived from *M. cajuputi* leaves were tested against *An. stephensi* larvae. The results clearly showed that the activity of the AchE enzyme decreased as the concentration of essential oil increased. The lowest activity of the AchE enzyme was found at a higher test concentration of 200 ppm/mL. When the larvae were exposed to essential oils, the activity of the acetylcholinesterase enzyme was not significant compared to the controls (from 12.33 to 7.0 mg protein/mL of homogenate) (Figure 4A). The enzyme level was found to be normal in the control larvae. In the treatment, the reduction of AchE enzymes in the larvae was primarily dose-dependent (see Figure 4A). Also, *M. cajuputi* essential oils treated An. stephensi larvae exhibited reduced levels of α and β-carboxylesterase enzyme activity (Figures 4B,C), reaching the lowest level within 24 h of treatment. The essential oils of *M. cajuputi* exhibited a dose-dependent effect on the enzyme activity of α-carboxylesterase in larvae. When the larvae were exposed to essential oils from *M. cajuputi*, their α-carboxylesterase activity was not significantly different compared to the controls (7.16–2.45 mg protein/mL of homogenate) (see Figure 4B). A similar finding was made with β-carboxylesterase, which showed a decrease in enzyme levels as the test concentration was increased (from 5.0 to 1.1 mM/protein/mg/min) in *An. stephensi* larvae (see Figure 4C). Similar to the present study, *Acacia nilotica* seed-derived essential oils showed not significant in acetylcholinesterase, α, and β-carboxylesterase (Perumal et al., 2023a).

According to the findings of the histological investigation, the larvae of *An. stephensi* were significantly impacted by their exposure to the essential oils derived from botanical sources. Cross-sections of *An. stephensi* larvae in their fourth instar that had been treated with essential oils caused lethal effects in the midgut tissues. The larval midgut of *An. stephensi* exhibited high damage, with distinct vacuolation within the epithelial cells of the midgut as well as in the adipose tissue and the muscles (Figure 3). These findings are comparable to *F. oxysporum* crude extracts is extremely toxic to the major disease mosquito vectors, which are *Aedes aegypti*, *An. stephensi*, and *Culex quinquefasciatus* (Vivekanandhan et al., 2018b).

Phytochemical analysis of essential oils derived from M. cajuputi reveals the presence of phenols, flavonoids, alkaloids, quinone, saponins, and tannins (Table 1). The phenols, flavonoids, alkaloids, quinone, saponins, and tannins derived from M. cajuputi essential oils may be involved in mosquito larvicidal activity (Table 1). Similar to the present study, Naringi crenulata (Rutaceae) plant extracts showed similar phytochemicals such as phenols, flavonoids, alkaloids, quinone, saponins, and tannins (Pratheeba et al., 2019). GC-MS analysis of M. cajuputi essential oils shows the presence of thirty-seven phytochemical constituents. Among the chemical constituents, the nine major are: 1,8-cineole (52.83%); limonene (13.50%); beta-pinene (5.43%); alpha-terpineol (4.65%); alpha-pinene (4.61%); gamma-terpinene (3.48); and beta-caryophyllene (3.11%). These major chemicals may have larvicidal activity (Table 2). Similar to the present study reported that the main chemical constituents of cajeput oil used in this study are the terpenes eucalyptol (44.86%), D-limonene (22.03%), and o-cymene (14.51%). There are an additional 8 minor chemical constituents in the cajeput oil, including γ-terpinene (7.87%), cyclofenchene (4.85%), α-terpineol (1.41%), α-phellandrene (1.01%), terpine 4-acetate (0.67%), β-pinene (0.66%), β-myrcene (0.65%), and (+)-4-carene (0.63%) (Rault et al., 2022). Similarly, Bakar et al. (2019) reported the main constituents of cajeput oil showed high mosquito larvicidal activity against dengue mosquito vectors *Aedes aegypti*.

Following the results of the bioassay was showed that essential oils derived from botanical sources did not have any impact on the *E. eugeniae* species. In the present study, essential oils derived from botanical sources showed the lower mortality rate ranged from 0% to 4%. On the other hand, the monocrotophos treatment served as a positive control, and it resulted in the death of more than 97.33% mortality of the earthworms (Figure 5; Figure 6). According to the findings, essential oils derived from plants do not have a significant adverse effect on earthworms because of their lower toxicity of chemical composition. Similar findings presented here, it was discovered that *Metarhizium anisopliae* conidia or secondary metabolites did not have any impact on *E. eugeniae* earthworm species when they were exposed to soil conditions (Perumal et al., 2023b). In addition, the chemical constituents derived from *M. anisopliae* did not cause pathogenicity in *E. eugeniae* when tested in a laboratory setting (Perumal et al., 2023c). Histological findings provide additional evidence in support of the essential oils derived from botanical sources that, in comparison to chemical pesticides (Monocrotophos), do not pose a threat to earthworm species that live in the soil (Figure 7). The findings demonstrated that essential oils derived from botanical sources do not cause pathogenicity within the gut tissues of earthworms. Previous studies have shown that extracts and essential oils derived from botanical sources do not have any toxic effects on the gut tissues of earthworms (Vivekanandhan et al., 2018a; Pratheeba et al., 2019; Perumal et al., 2023a). This current result lends support to the findings of those earlier studies. The findings demonstrated that all of the earthworm tissues, including the epidermis, circular muscle, setae, mitochondrion, and intestinal lumen tissues, were normal and comparable to the control group. On the other hand, monocrotophos appeared to be completely toxic to the tissues of earthworms.

## Conclusion

Plant essential oil compositions continue to be a promising source of natural product chemistries that have the potential to reduce populations of mosquitoes that transmit disease-causing pathogens and, ultimately, to mitigate the transmission of human diseases within and between communities. In order to develop and implement effective bioinsecticides, it is necessary to first determine the chemistries that interact with essential oils (EO) and the mechanism by which they exert their effects. This study is the first-ever report on the biological insecticidal activity of cajeput oil against the mosquito vector *An. stephensi*, which is responsible for transmitting the malarial disease. It is necessary to conduct additional research in order to provide conclusive evidence that the essential oil receptor modulation is the mechanism of action that is responsible for the biological insecticidal activity of cajeput oil.

## Data Availability

The original contributions presented in the study are included in the article/Supplementary material, further inquiries can be directed to the corresponding author.
